# Nek2 augments sorafenib resistance by regulating the ubiquitination and localization of β-catenin in hepatocellular carcinoma

**DOI:** 10.1186/s13046-019-1311-z

**Published:** 2019-07-18

**Authors:** Ling Deng, Jingyuan Sun, Xiaohui Chen, Li Liu, Dehua Wu

**Affiliations:** 1 Department of Radiation Oncology, Nanfang Hospital, Southern Medical University, Guangzhou, 510515 China; 2 Hepatology Unit and Department of Infectious Diseases, Nanfang Hospital, Southern Medical University, 1838 Guangzhoudadaobei Road, Guangzhou, Guangzhou, 510515 China

**Keywords:** Hepatocellular carcinoma, Sorafenib, Drug resistance, Nek2, β-Catenin

## Abstract

**Background:**

Sorafenib is the first-line treatment for advanced-stage hepatocellular carcinoma (HCC). Several studies have shown that the up-regulation of β-catenin plays a role in sorafenib resistance in HCC; however, the mechanism associated with this phenomenon remains elusive.

**Methods:**

Western blotting, flow cytometry, and an evaluation of IC_50_ values were used to confirm the role of β-catenin in HCC sorafenib resistance. Immunoprecipitation and western blotting were then performed to identify regulatory interactions between β-catenin and Nek2. Further, western blotting, flow cytometry, and an in vivo xenograft model were used to evaluate the function of Nek2 in HCC sorafenib resistance, whereas rescue experiments were performed to confirm that Nek2 induces sorafenib resistance via β-catenin. Finally, western blotting and immunohistochemistry were used to evaluate the expression level of Nek2 in paired HCC and non-tumor tissues.

**Results:**

We showed that β-catenin could suppress sorafenib-induced apoptosis and cell growth inhibition in HCC cell lines. By screening β-catenin-interacting proteins, we found that Nek2 could bind β-catenin in sorafenib-treated HCC cell lines. Our results also showed that Nek2 stabilizes β-catenin and promotes its translocation to the nucleus, consequently activating the transcription of downstream target genes. We further confirmed that Nek2 could induce sorafenib resistance in HCC cell lines, and that β-catenin was the key element involved in this process. Further, a xenograft tumor model showed that Nek2 knockdown could improve the anti-tumor effect of sorafenib, whereas an analysis of tumor proteins showed that Nek2 regulates β-catenin protein levels and its nuclear translocation in vivo. In addition, Nek2 was found to be up-regulated in HCC tissue, and especially in advanced-stage disease.

**Conclusions:**

Our study proves that Nek2 induces HCC sorafenib resistance via β-catenin and suggests a novel therapeutic strategy to improve the anti-tumor effects of sorafenib in HCC.

**Electronic supplementary material:**

The online version of this article (10.1186/s13046-019-1311-z) contains supplementary material, which is available to authorized users.

## Background

Liver cancer is one of the greatest threats to human health worldwide, accounting for 4.7% of newly diagnosed cancer cases and 8.2% of deaths caused by cancer in 2018 [1]. Hepatocellular cancer (HCC) comprises 90% of liver cancer cases [2, 3]. China is one of the most high-risk regions for HCC, as approximately 420,000 people die from this disease in this country every year [4]. Despite efforts to elucidate the mechanisms associated with HCC and the burden it caused [5, 6], the understanding of this disease is still limited and treatment options are not optimal. Sorafenib, a multi-target tyrosine kinase inhibitor, was the only systemic therapy for advanced-stage HCC patients from 2007 to 2016 and it remains the first-line treatment for these patients [7]. Although sorafenib is associated with overall survival benefits for advanced-stage HCC patients, the response rate is not yet satisfactory [8, 9]. Thus, a systematic understanding of the mechanisms associated with sorafenib resistance is critical to improve treatment options for HCC patients.

Wnt/β-catenin signaling plays essential roles in multiple physiological and pathological processes [10, 11]. Studies have revealed that this pathway regulates the initiation and development of many types of cancer [12, 13]. For example, in multiple myeloma, the inhibition of canonical Wnt signaling suppresses tumor growth and sensitizes cells to anti-tumor drugs [14]. Further, in breast cancer, inhibition of β-catenin by overexpressing LINC00968 was fond to reduce drug resistance [15]. There have also been several studies showing that the upregulation of β-catenin is related to sorafenib resistance [16, 17] and that inhibiting Wnt/β-catenin signaling can increase the efficacy of this drug [18]. However, the mechanism underlying the dysregulation of β-catenin during sorafenib resistance is still inconclusive, and thus, more studies are required to improve the efficacy of this agent.

We performed this study to identify potential regulators of β-catenin during sorafenib resistance in HCC. Our results demonstrated that NIMA-related kinase 2 (Nek2) binds β-catenin, blocking the interaction between this protein and the destruction complex, and ultimately contributing to sorafenib resistance for HCC. Thus, the genetic manipulation of Nek2 could influence the efficacy of sorafenib in HCC, both in vitro and in vivo.

## Materials and methods

### Plasmid, small interfering RNA lentivirus and adenovirus

Full-length Nek2 and β-catenin were cloned in pFlag-CMV2 and Nek2 also in pEGFP-N1. The construction of Nek2 mutant plasmid was using KOD -Plus- Mutagenesis Kit (TOYOBO, Japan) base on Flag-Nek2 according to manufacture’s instruction. The Nek2 siRNA target sequences are as follow: 5′-GUCCUACAAUGAGAAAUCATT -3′; 5′- GAGACUAGCAGAGGACAAATT-3′.

The β-catenin siRNA target sequences are as follow: 5′-GGAUGUGGAUACCUCCCAATT-3′; 5′- GAUGGUGUCUGCUAUUGUATT-3′. For lentivirus Nek2 overexpression and knockdown, full-length Nek2 was cloned in GV438 and two specifically targeting Nek2 hairpin precursors (5′-AGACGAGCAAAGAAGAAAT-3′ and 5′-TTACTCTGATGAATTGAAT-3′) were inserted into GV112. Adenovirus containing full-length β-catenin is purchased from Vigene Biosciences, USA (VH800675).

### Cell lines culture and transfection

SMMC-7721, MHCC-97H, SK-Hep1 and HEK-293 T were obtained from the Cell Bank of Type Culture Collection (CBTCC, Chinese Academy of Sciences, Shanghai, China). Cells were maintained at 37 °C in a humidified incubator containing 5% CO_2_ in DMEM and RPMI-1640 (Invitrogen, USA) supplemented with 10% fetal bovine serum (Gibco, USA). The expression plasmids were transfected into HCC cell lines using Lipofectamine 3000 (Invitrogen, USA) and siRNA using Lipofectamine RNAiMAX Reagent (Invitrogen, USA) according to manufacture’s instruction.

### Clinical samples

Fresh HCC samples and the paired adjacent noncancerous tissues were collected from 29 patients who underwent initial hepatectomy between January 2011 and July 2012 at Nanfang Hospital, Southern Medical University (Guangzhou, China). Another 102 paraffin-embedded tissues from the patients with HCC, who underwent initial hepatectomy between January 2006 and July 2009 at the same hospital and were followed for 5 years, were selected from the Department of Pathology. All the patients were not pretreated with chemotherapy or radiotherapy before the surgery.

### RNA isolation and quantitative real-time PCR (qRT-PCR)

Total RNA was extracted using TRIzol Reagent (TAKARA, Dalian, China). Reverse transcription was processed using Prime RT reagent Kit (TAKARA, Dalian, China). Quantitative Real-Time PCR (qRT-PCR) was performed using SYBR® Premix Ex Taq™ Kit (TAKARA) according to the manufacturer’s instructions. The sequences for the forward and reverse primers of β-actin are as follows: forward 5′- CCGTTCCGAAAGTTGCCTTTT-3′ and reverse 5′- ATCATCCATGGTGAGCTGGC-3′. Primers specific for Nek2: forward 5′-AGATTGGAGCAGAAAGAACAGGAGC-3′ and reverse 5′-TGCACTTGGACTTAGATGTGAGCTG-3′. Primers specific for β-catenin: forward 5′- GCGCCATTTTAAGCCTCTCG − 3′ and reverse 5′- AAATACCCTCAGGGGAACAGG-3′. Primers specific for c-Myc: forward 5′- CAGCGACTCTGAGGAGGAAC-3′ and reverse 5′- CTGGTGCATTTTCGGTTGT-3′. Primers specific for CyclinD1: forward 5′- GGGCAGCAGAAGCGAGAG − 3′ and reverse 5′- GTTCCTCGCAGACCTCCAG − 3′.

The relative quantification was calculated as following: ΔCt [ΔCt = Ct (Nek2) - Ct (β-actin)]. Relative expression level was determined as 2^-ΔΔCt^, where ΔΔCt = ΔCt (test samples) -ΔCt (reference samples).

### Antibodies and reagents

Antibodies used in this study are as follow: β-catenin, PARP, Caspase3, Bcl-2, Bax, β-actin, CyclinD1, c-Myc were purchased from Proteintech Group, UK. Nek2 was purchased from BD Biosciences. Survivin was purchased from CST. Flag and HA were purchased from Sigma-Aldrich.

Sorafenib(S7397) and TAI-1(S7495) was purchased from selleck, USA. Cycloheximide and MG132 were purchased from Sigma-Aldrich.

### Immunoprecipitation

For co-immunoprecipitation, cells were transfected with indicated plasmid. 48 h after transfection, cells were extracted in IP lysis buffer and incubated for 30 min. The lysate was cleared by 12,000 rpm centrifugation for 15 min. One milligrams of protein from each group was mixed with indicated antibody or IgG as negative control and incubated at 4 °C overnight, then 30 μl of protein A and G agarose were added into the mixture. After 3 h of incubation, collected the agarose and washed it with IP lysis buffer for 5 times and denatured the sample.

### Western blotting assay

The quantified protein samples were separated by electrophoresis on a SDS-polyacrylamide gel before transfer onto polyvinylidene fluoride (PVDF, Millipore, Bedford, MA, USA) membranes. After the membranes were blocked with 5% BSA for 1 h in room temperature, they were incubated with specific antibody, followed by the secondary antibody conjugated to horseradish peroxidase. Next, the signals of the membranes were detected by ECL(enhanced chemiluminescence)Western Blotting Substrate (Pierce, Rockford, IL) according to the manufacturer’s instructions. The band intensity of western blotting and the normalization were analyzed using the Image J program (Tree Star lnc., Ashland, OR).

### Immunofluorescence

SMMC-7721 cells transfected with Flag-cmv2 or Flag-Nek2 were planted on coverslips for immunofluorescence staining. 48 h after transfection, cells were washed twice by PBS, fixed in 4% paraformaldehyde. 10 min later, cells were fixed on coverslips and washed by PBS. Cells were permeabilized with 0.25% Triton X-100 for 7 min, and 1 h incubation with 5% BSA in room temperature. Cells were further incubated with Flag antibodies (1:800) and β-catenin antibodies (1:100) in 4 °C overnight, washed by PBS, and incubated with mixture containing FITC-conjugated CA goat antibodies against mouse IgG and Cy3-conjugated CA goat antibodies against rabbit IgG (Santa Cruz Biotechnology, Santa Cruz, CA, USA) for 1 h in room temperature. The coverslips were counterstained with 4, 6-diamidino-2-phenylindole (Invitrogen) and imaged with a confocal laser-scanning microscope (Carl Zeiss LSM880 confocal microscope, Germany).

### Cell proliferation and apoptosis assay

Cell proliferation analysis was detected with CCK-8 Kit (Dojindo, Japan). Cells were seeded in 96-well plates with indicated transfection, and the CCK-8 proliferation assay was tested as manufacturer’ s instructions. Cell apoptosis was detected with Annexin V-FITC Kit (Absin) using flow cytometric analysis.

### Animal experiments

Male nude mice aged 4-week were subcutaneously injucted with 1*10^7^ MHCC-97H or cells transfected with shRNA control or shNek2. Tumors were measured every two days and the tumor volume was defined as (length*width^2^)/2. When the mean tumor volume reached approximately 100mm^3^, mice were divided into indicated groups. Sorafenib was intragastric administrated at a dose of 30 mg/kg/day for 14 days and TAI-1 was intraperitoneal injection at a dose of 20 mg/kg/day for 14 days, synchronized with sorafenib. The mice were then sacrificed at the end of treatments.

### Immunohistochemistry

IHC staining was carried out using Dako Envision System (Dako, Carpinteria, CA) according to the manufacturer’s protocol. The IHC-stained tissue sections were scored by two pathologists who were blinded to the clinical parameters. The scores of staining intensity were defined as following: 0 (negative), 1 (weak), 2 (medium), and 3 (strong). The extent of staining was scored as 0 (0%), 1 (1–25%), 2 (26–50%), 3 (51–75%), and 4 (76–100%), according to the percentages of the positive staining areas in relation to the entire carcinoma-involved area or the entire section for the normal samples. we regarded the sum of the intensity and extent scores as the final staining score (0–7) for Nek2. For the purpose of statistical evaluation, tumors with a final staining score of >4 were considered as highly Nek2 expressing.

### Statistical analysis

All statistical analysis was performed using the SPSS statistical software version 22 (Abbott Laboratories, North Chicago, IL).Student’s t-test and one-way ANOVA test were performed for comparing differences. The relationship between Nek2 expression and various clinicopathological parameters was evaluated with the Mann-Whitney U-tests. Survival curves were plotted by the Kaplan-Meier method and compared by the log-rank test. The effects of variables on survival were determined by univariate and multivariate Cox proportional hazards model. *P*-value < 0.05 was considered significant.

## Results

### β-Catenin/Wnt signaling induces sorafenib resistance in hepatocellular carcinoma

**Fig. 1:**
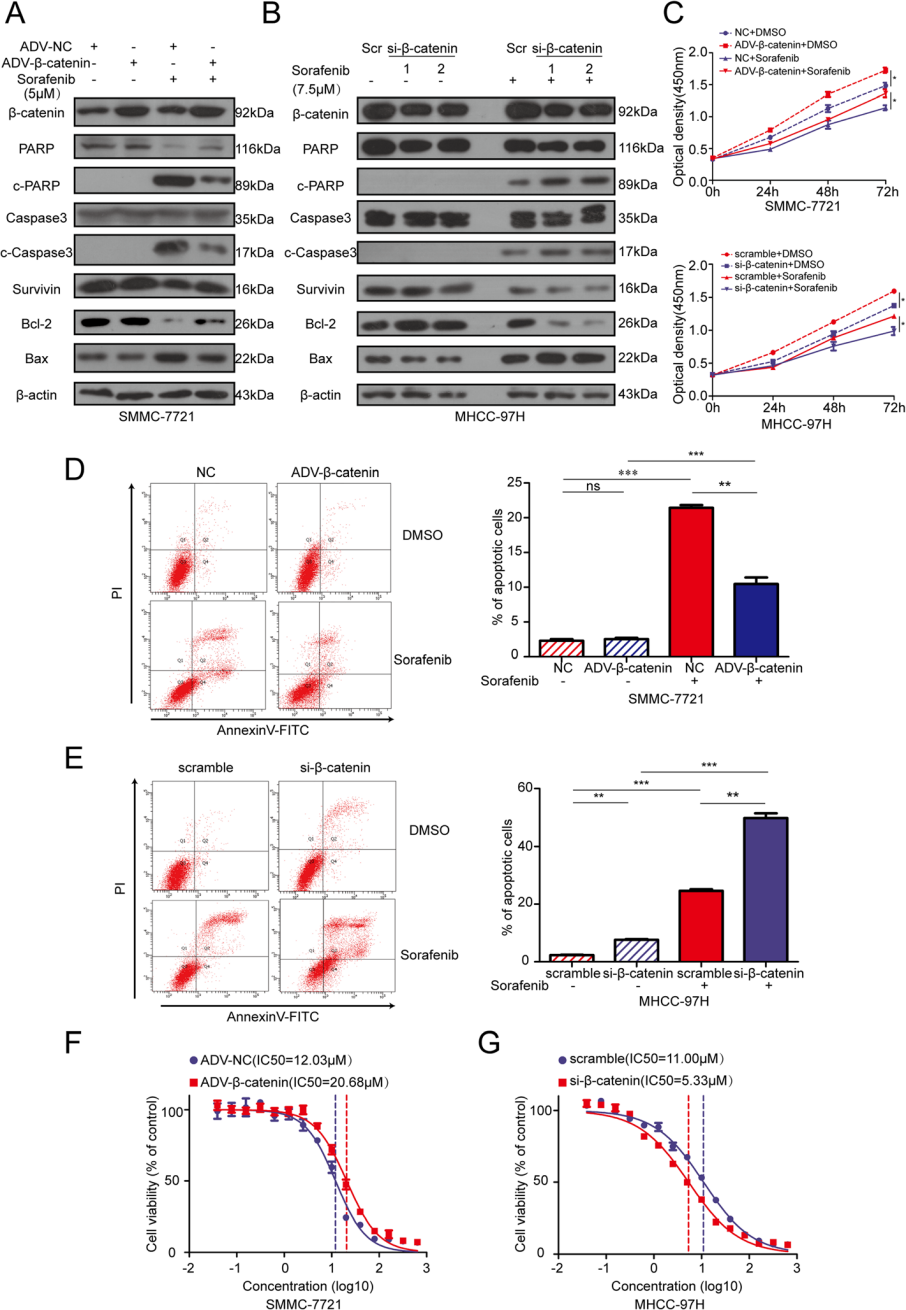
β-catenin suppressed cells apoptosis and growth inhibition induced by sorafenib treatment in HCC cell lines. **a**. SMMC-7721 cells were transfected with adenovirus for control or overexpressing β-catenin. 24 h after transfection cells were treated with sorafenib (5 μM) for another 24 h. Cells were collected for western blotting assay. **b**. MHCC-97H cells were transfected with siRNA for scramble or β-catenin for 24 h and sorafenib (7.5 μM) treatment for another 24 h before western blotting assay. **c**. CCK-8 assays were performed to detect the growth inhibition of sorafenib on SMMC-7721 (upper panel) and MHCC-97H (bottom panel) with ADV-β-catenin or si-β-catenin. **d**, **e**. (Left panels) SMMC-7721 and MHCC-97H cells with overexpressing or silencing β-catenin were treated with sorafenib and cells were analyzed by flow cytometry. (Right panels) Columns, representing the total percentage of Q2 and Q4, were the average of three independent experiments. **f**, **g**. Dose-dependent effects of sorafenib on the viability of SMMC-7721 (**f**) and MHCC-97H (**g**) with ADV-β-catenin or si-β-catenin. Data were presented as mean ± SEM, ns, no significance; *P<0.05; **P<0.01; ***P<0.001

Consist with previous studies, our experiment also showed that after 24 h of sorafenib treatment, levels of β-catenin and its downstream target genes including *c-Myc* and *CyclinD1* were upregulated in SMMC-7721, MHCC-97H, and SK-Hep1 HCC cell lines (Additional file 1: Figure S1a). To further confirm the role of β-catenin in sorafenib resistance, we first used adenovirus to overexpress it in the SMMC-7721 HCC cell line, and cells were subsequently treated with sorafenib and harvested for protein analysis. Western blotting showed that in response to this treatment, the overexpression of β-catenin resulted in a significant decrease in the levels of pro-apoptotic proteins including cleaved-PARP, cleaved-caspase-3, and Bax, but increased anti-apoptotic proteins including Bcl-2 and survivin (Fig. 1a). We next determined if β-catenin silencing could enhance sorafenib efficacy in HCC cell lines. The introduction of two different si-β-catenin sequences into MHCC-97H and SK-Hep1 cells significantly increased pro-apoptotic protein expression and decreased anti-apoptotic protein expression in response to sorafenib treatment (Fig. 1b and Additional file 1: Figure S1b). Furthermore, CCK-8 proliferation assays showed that sorafenib could significantly suppress the growth of SMMC-7721, MHCC-97H, and SK-Hep1 cells (Fig. 1c and Additional file 1: Figure S1c). However, overexpressing β-catenin in SMMC-7721 cells ameliorated these inhibitory effects of sorafenib, whereas silencing β-catenin in MHCC-97H and SK-Hep1 cells enhanced such effects (Fig. 1c and Additional file 1: Figure S1c). Similarly, flow cytometric analysis also showed that the overexpression of β-catenin could decrease sorafenib-induced cell apoptosis (Fig. 1d), whereas β-catenin silencing enhanced this effect (Fig. 1e and Additional file 1: Figure S1d). We also tested the IC_50_ of sorafenib in HCC cell lines with β-catenin overexpression or knockdown. Overexpressing β-catenin was found to increase the IC_50_ in SMMC-7721 cells (Fig. 1f), whereas IC_50_ values were lower in MHCC-97H and SK-Hep1 cells treated with si-β-catenin (Fig. 1g and Additional file 1: Figure S1e). Taken together, these results suggest that β-catenin and the activation of Wnt signaling are closely correlated with sorafenib resistance in HCC.

### Nek2 binds β-catenin and activates target gene transcription

**Fig. 2:**
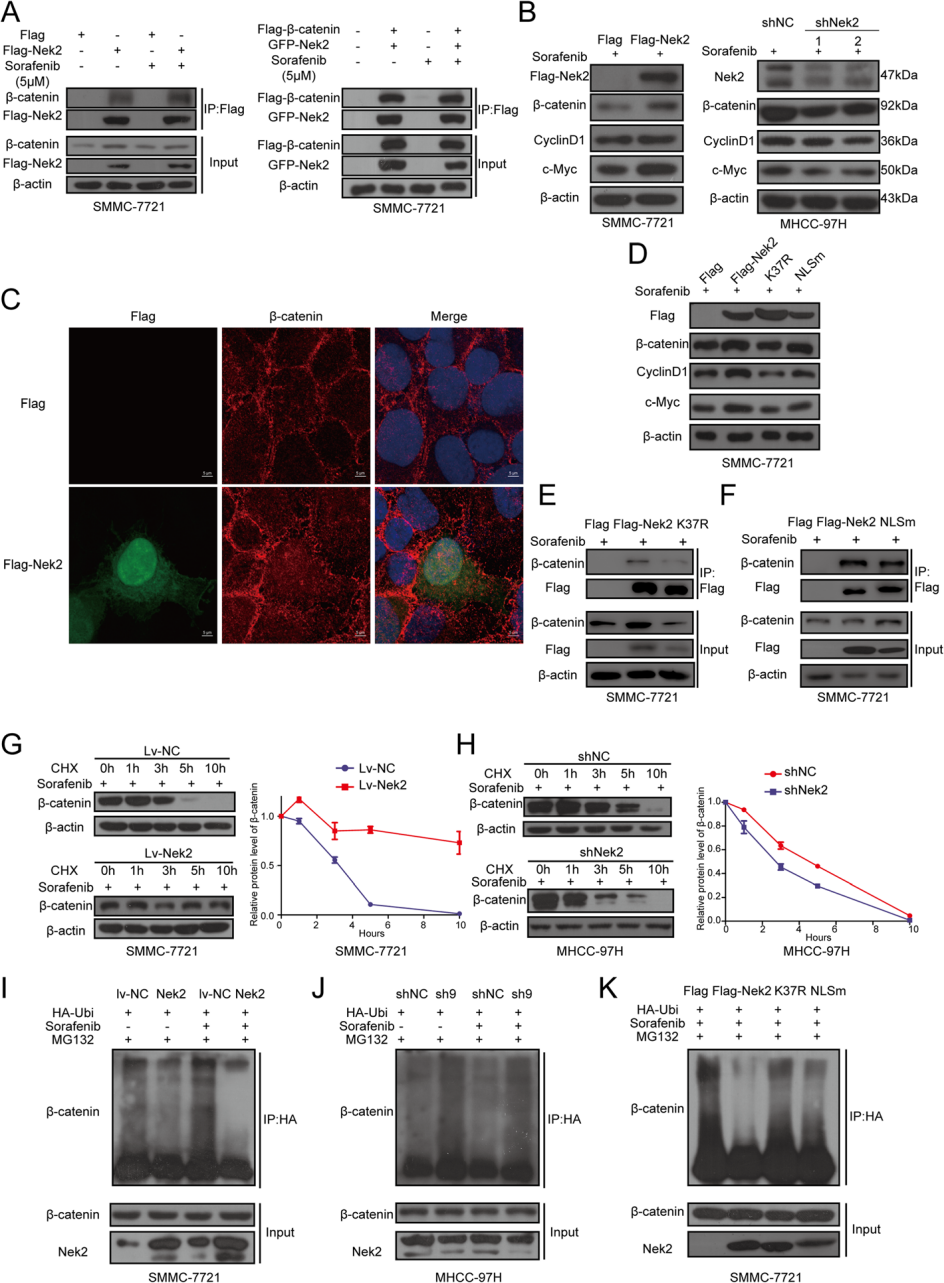
Nek2 bond β-catenin and regulated ubiquitination and nuclear translocation of β-catenin in HCC cell lines. **a**. SMMC-7721 cells were transfected with indicated plasmids with or without sorafenib treatment. Flag-tagged protein were precipitated and associated protein were detected with western blotting assay. **b**. SMMC-7721 cells, transfected with Flag-cmv2 or Flag-Nek2 plasmids, and MHCC-97H cells, transfected with lentivirus containing shRNA for Nek2 were treated with sorafenib for 24 h and analyzed with western blotting assay. **c**. Flag-Nek2 (green) and β-catenin (red) were detected using immunofluorescence assay. **d**. SMMC-7721 cells transfected with indicated plasmids for 24 h and sorafenib treatment for another 24 h, then were analyzed with western blotting assay. **e**, **f**. SMMC-7721 cells were transfected with Flag, Flag-Nek2, mutation plasmids with sorafenib treatment. Flag-tagged protein were precipitated and associated protein were monitored with western blotting assay. **g**, **h**. (Left panels) SMMC-7721 or MHCC-97H stable overexpression or knockdown of Nek2 first treated with sorafenib for 24 h, then treated with CHX (10 μM) and collected cells at indicated timings. (Right panels) Time line charts for β-catenin gray-scale at different timings normalized to β-actin gray-scale. i, j, k. SMMC-7721 and MHCC-97H cells were co-transfected with indicated lentivirus or plasmids with His-tagged ubiquitin in the indicated combinations. Cells were changed with medium containing MG132 (20 μM) 6 h before harvested and analyzed with western blotting assay

The findings described in the previous section prompted us to investigate the regulation of β-catenin in HCC and during sorafenib resistance. We searched BioGRID (https://thebiogrid.org/) to explore potential β-catenin-interacting proteins. We also used GSE62813 and GSE74666 datasets, which contain information on expression profile changes in cancer cells after sorafenib treatment, to help us identify such interacting proteins related to sorafenib resistance. Among the proteins that were thought to interact with β-catenin, we found that Nek2 could bind β-catenin at centrosomes to regulate the centrosome cycle during mitosis [19]. We also noticed that Nek2 was up-regulated after sorafenib treatment based on GSE62813 and GSE74666 datasets. Hence, we decided to explore the possible relationship between Nek2 and β-catenin in sorafenib-treated HCC cells. To probe the mechanisms underlying the regulatory interaction between Nek2 and β-catenin in response to sorafenib treatment, the specific binding of these two proteins was tested by immunoprecipitation analysis in SMMC-7721, MHCC-97H, and HEK-293 T cell lines (Fig. 2a and Additional file 2: Figure S2a, b). Interestingly, we observed that when cells expressed high levels of Nek2, the interaction between Nek2 and β-catenin was associated with a diminished interaction between β-catenin and GSK3β, which is an important member of the destruction complex (Additional file 2: Figure S2a). These findings suggest that Nek2 interferes with the interaction between β-catenin and the destruction complex. Since Nek2 was found to bind β-catenin in response to sorafenib treatment, we assumed whether Nek2 could regulate β-catenin expression levels. Our results demonstrated that after sorafenib treatment, Nek2 overexpression increased β-catenin protein levels and Wnt/β-catenin targets including CyclinD1 and c-Myc, whereas Nek2 silencing decreased β-catenin protein levels and downstream target genes (Fig. 2b and Additional file 2: Figure S2c). Meanwhile, qPCR analysis showed that Nek2 did not alter the mRNA levels of *β-catenin*, whereas changes in the mRNA expression of Wnt pathway target genes were consistent with protein levels (Additional file 2: Figure S2d–f). These results suggest that Nek2 regulates β-catenin protein levels through post-translational modification. Furthermore, immunofluorescence showed that the overexpression of Nek2 in SMMC-7721 cells apparently increased the nuclear translocation of β-catenin, as compared to that in cells transfected with an empty vector (Fig. 2c). To further demonstrate the translocation of β-catenin, we separately extracted proteins from cytoplasmic and nuclear compartments. Western blotting showed that β-catenin levels in the nucleus were significantly increased in Nek2-overexpressing SMMC-7721 cells, but were decreased in Nek2-silenced MHCC-97H cells (Additional file 2: Figure S2 g, h). These findings indicate that Nek2 can bind β-catenin, promote its nuclear translocation, and activate Wnt/β-catenin target gene transcription in response to sorafenib treatment.

To determine if the interaction between Nek2 and β-catenin is related to the kinase activity of Nek2, we constructed a kinase-dead mutant of Nek2 (K37R; Additional file 3: Figure S3a). Western blotting showed that compared to those with wild-type Nek2, the K37R variant could not increase protein levels of β-catenin and its downstream target genes (Fig. 2d). Co-immunoprecipitation showed that the interaction between K37R and β-catenin was also diminished (Fig. 2e). These findings indicate that Nek2 kinase activity is crucial for the regulation of β-catenin. At the same time, we assumed whether cytoplasmic Nek2 was sufficient to regulate β-catenin, and thus, we constructed a nuclear localization sequence mutant of Nek2 (NLSm; Additional file 3: Figure S3a); using this, we confirmed that this mutant could still interact with β-catenin and regulate its activity (Fig. 2d, f). When we analyzed relative protein levels, we noticed that even though NLSm could increase β-catenin levels, similar to that observed with the wild-type protein, downstream target genes were not up-regulated to the same extent (Fig. 2d). Further experiments showed that the protein levels of β-catenin in the nuclear extract were not increased significantly in NLSm-overexpressing SMMC-7721 cells (Additional file 3: Figure S3b). These results suggest that Nek2 not only protects β-catenin from the destruction complex in the cytoplasm but also escorts it to the nucleus.

### Nek2 regulates β-catenin ubiquitination and protects it from proteasome degradation`

Previously described data herein suggested that Nek2 regulates β-catenin by blocking its proteasomal degradation. To verify this hypothesis, we inhibited the function of the proteasome using MG132, a classic proteasome inhibitor, in MHCC-97H cells transfected with lentiviral particles expressing Nek2-targeting shRNA. Results showed that silencing Nek2 no longer resulted in decreased β-catenin protein levels in the presence of MG132 (Additional file 3: Figure S3c). We next blocked protein synthesis using cycloheximide in SMMC-7721 and MHCC-97H cells, which have different Nek2 levels. We observed that with sorafenib treatment, the half-life of β-catenin was increased in SMMC-7721 cells overexpressing Nek2 (Fig. 2g) and was decreased in Nek2-knockdown MHCC-97H cells (Fig, 2 h), indicating that Nek2 stabilizes β-catenin. This result was also confirmed in SK-Hep1 cells treated with si-Nek2. Similarly, the overexpression of NLSm Nek2 increased the half-life of β-catenin (Additional file 3: Figure S3e). Since ubiquitination directs β-catenin to the proteasome for degradation, we tested whether the Nek2-regulatory effect was due to the ubiquitination of β-catenin. Indeed, when we overexpressed Nek2 in SMMC-7721 cells, β-catenin ubiquitination was significantly reduced (Fig. 2i), whereas silencing Nek2 in MHCC-97H cells significantly increased the ubiquitination of β-catenin (Fig. 2j). Interestingly, we observed that with sorafenib treatment, the regulatory effect on β-catenin ubiquitination was more pronounced. Comparing the effects of wild-type and mutant Nek2, we observed that the K37R variant could not affect the ubiquitination of β-catenin and that NLSm also resulted in decreased β-catenin ubiquitination (Fig. 2k). Taken together, these results suggest that Nek2 stabilizes β-catenin by regulating its ubiquitination.

### Nek2 induces sorafenib resistance in HCC cells

**Fig. 3:**
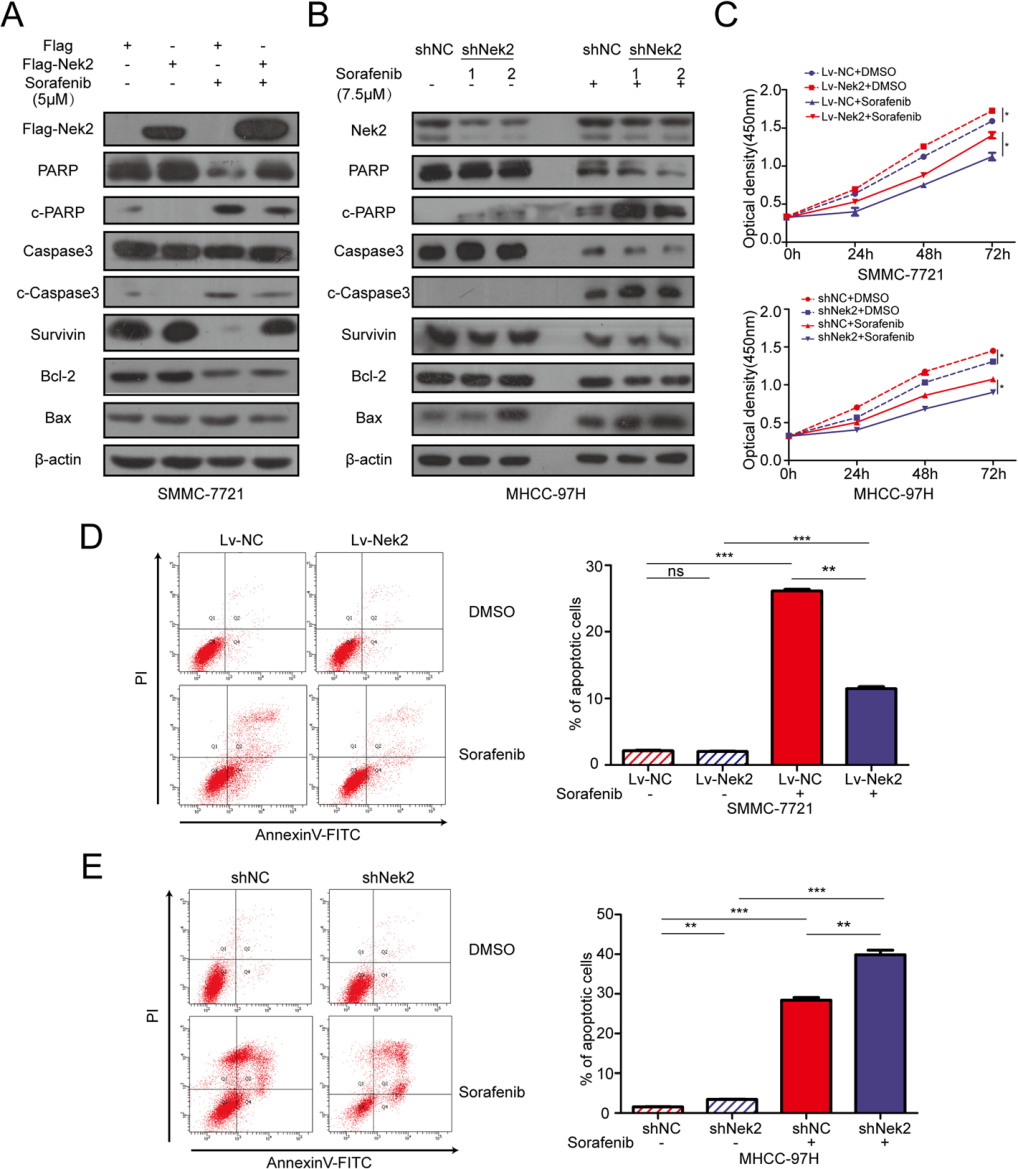
Nek2 induced sorafenib resistance in HCC cell lines. **a**, **b**. SMMC-7721 and MHCC-97H cells transfected with Nek2 plasmids or sh-Nek2 were treated with sorafenib for 24 h and analyzed with western blotting assay for pro-apoptotic and anti-apoptotic proteins. **c**. CCK-8 assays were performed to detect the growth inhibition of sorafenib on SMMC-7721 (upper panel) and MHCC-97H (bottom panel) with lentivirus Nek2 or sh-Nek2. **d**, **e**. (Left panels) SMMC-7721 and MHCC-97H cells were treated with sorafenib with lentivirus overexpression or knockdown of Nek2 and cells were analyzed by flow cytometry. (Right panels) columns, representing the total percentage of Q2 and Q4, were the average of three independent experiments. Data were presented as mean ± SEM, ns, no significance; *P<0.05; **P<0.01; ***P<0.001

Given the effects on β-catenin regulation by Nek2 and the role of β-catenin in sorafenib resistance, we hypothesized that Nek2 could also induce sorafenib resistance in HCC. Indeed, western blotting showed that overexpression of Nek2 decreased the levels of cleaved-PARP, cleaved-caspase-3, and Bax upon sorafenib treatment, but increased Bcl-2 and survivin (Fig. 3a). Consistently, silencing Nek2 using shRNA increased the level of pro-apoptotic proteins and decreased the level of anti-apoptotic proteins (Fig. 3b and Additional file 4: Figure S4a). CCK-8 proliferation assays also showed that the inhibitory effect of sorafenib on cell growth was decreased in SMMC-7721 cells with higher levels of Nek2 but was increased in MHCC-97H and SK-Hep1 cells with lower levels of Nek2 (Fig. 3c and Additional file 4: Figure S4b). Similarly, using flow cytometry to measure the ratio of apoptotic cells, we showed that Nek2 overexpression could decrease the proportion of apoptotic cells after sorafenib treatment (Fig. 3d), and that silencing Nek2 increased sorafenib-induced apoptosis (Fig. 3e and Additional file 4: Figure S4c). Overall, these results indicate that Nek2 induces sorafenib resistance in HCC cell lines.

### Nek2 induces sorafenib resistance via β-catenin

**Fig. 4:**
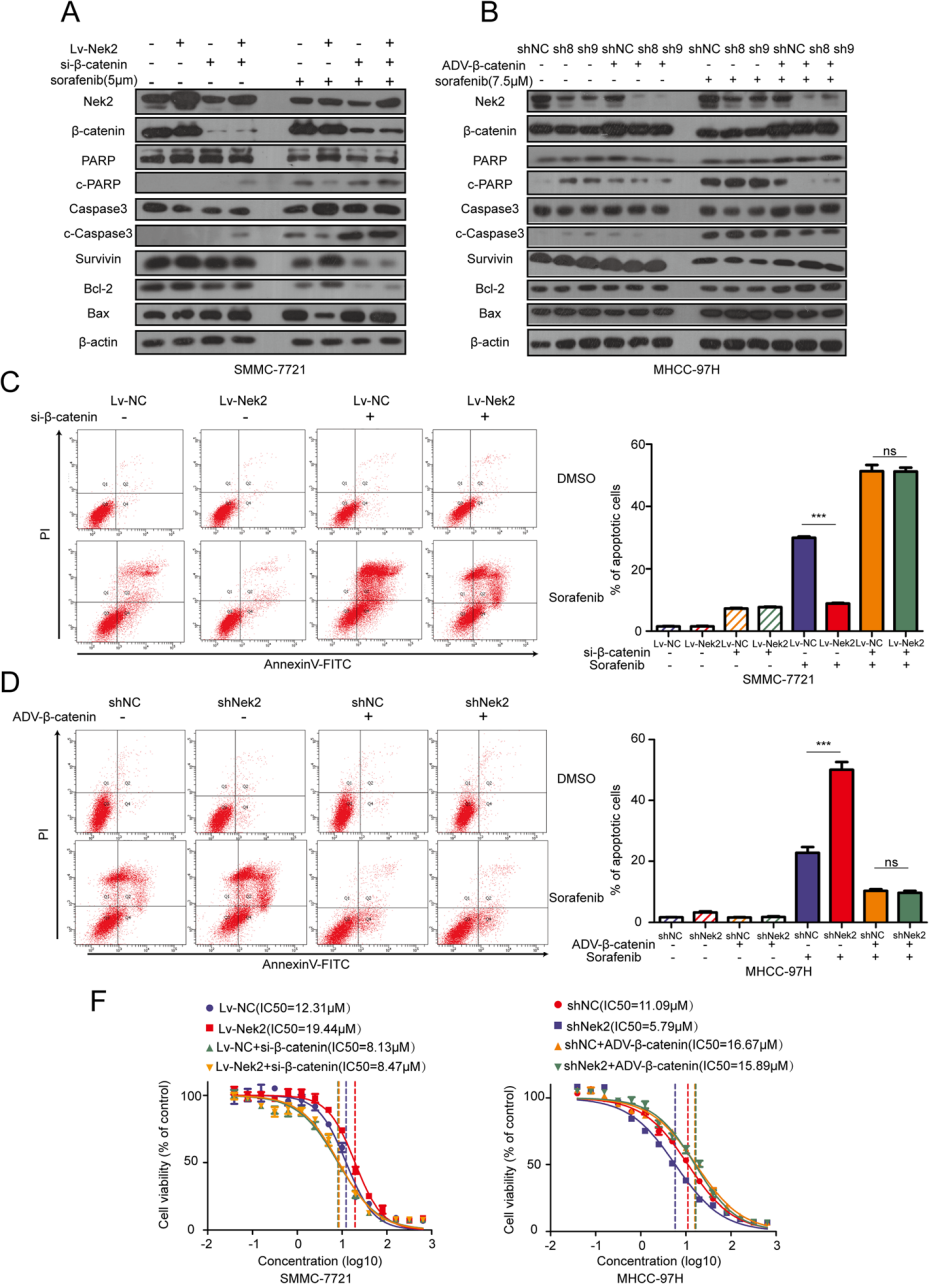
Nek2 induced sorafenib resistance via β-catenin in HCC cell lines. **a**. SMMC-7721 transfected with different combinations of lentivirus Nek2 and si-β-catenin were treated with or without sorafenib and cells were analyzed using western blotting assay. **b**. MHCC-97H transfected with different combinations of shRNA Nek2 and adenovirus-β-catenin were treated with or without sorafenib and cells were analyzed using western blotting assay. **c**, **d**. (Left panels) Flow cytometry was used to detect the apoptotic cells in different combinations indicated. (Right panels) columns, representing the total percentage of Q2 and Q4, were the average of three independent experiments. Data were presented as mean ± SEM, ns, no significance; *P<0.05; **P<0.01; ***P<0.001. f. Dose-dependent effects of sorafenib on the viability of SMMC-7721 (left panel) and MHCC-97H (right panel) with indicated combination of Nek2 and β-catenin

To determine whether β-catenin participates in Nek2-induced sorafenib resistance in HCC cells, western blotting was first performed to analyze apoptosis. This showed that in SMMC-7721 cells with Nek2 overexpression, β-catenin knockdown could reverse the decrease in pro-apoptotic protein levels and the increase in anti-apoptotic proteins in response to sorafenib treatment (Fig. 4a). Consistently, the overexpression of β-catenin altered pro- and anti-apoptotic protein levels upon sorafenib treatment in MHCC-97H and SK-Hep1 cells with knockdown of Nek2 (Fig. 4b and Additional file 5: Figure S5a). An examination of the ratio of apoptotic cells, as measured by flow cytometry, also showed that the overexpression of Nek2 combined with β-catenin silencing could not mitigate the increased in the proportion of apoptotic cells induced by sorafenib (Fig. 4c). Nek2 silencing with β-catenin overexpression was also not able to abrogate the enhanced level apoptosis ratio induced by sorafenib in MHCC-97H and SK-Hep1 cells (Fig. 4d and Additional file 5: Figure S5b). To further determine whether Nek2 can mediate resistance to sorafenib in HCC by modulating β-catenin, we determined its IC_50_ in SMMC-7721, MHCC-97H, and SK-Hep1 with different expression levels of Nek2. In SMMC-7721, MHCC-97H, and SK-Hep1 cells, these values were 12.31, 11.09, and 6.40 μM, respectively. However, in SMMC-7721 cells overexpressing Nek2 this increased to 19.44 μM, whereas silencing β-catenin decreased this value to 8.47 μM. The IC_50_ value for MHCC-97H cells treated with sh-Nek2 was 5.79 μM, whereas β-catenin overexpression increased this value to 15.89 μM (Fig. 4f). In SK-Hep1 cells the corresponding IC_50_ values were 3.61 and 9.77 μM, respectively (Additional file 5: Figure S5c). These data indicated that Nek2 can induce sorafenib resistance in HCC cells by regulating β-catenin.

### Nek2 knockdown improves sorafenib efficiency in vivo

**Fig. 5:**
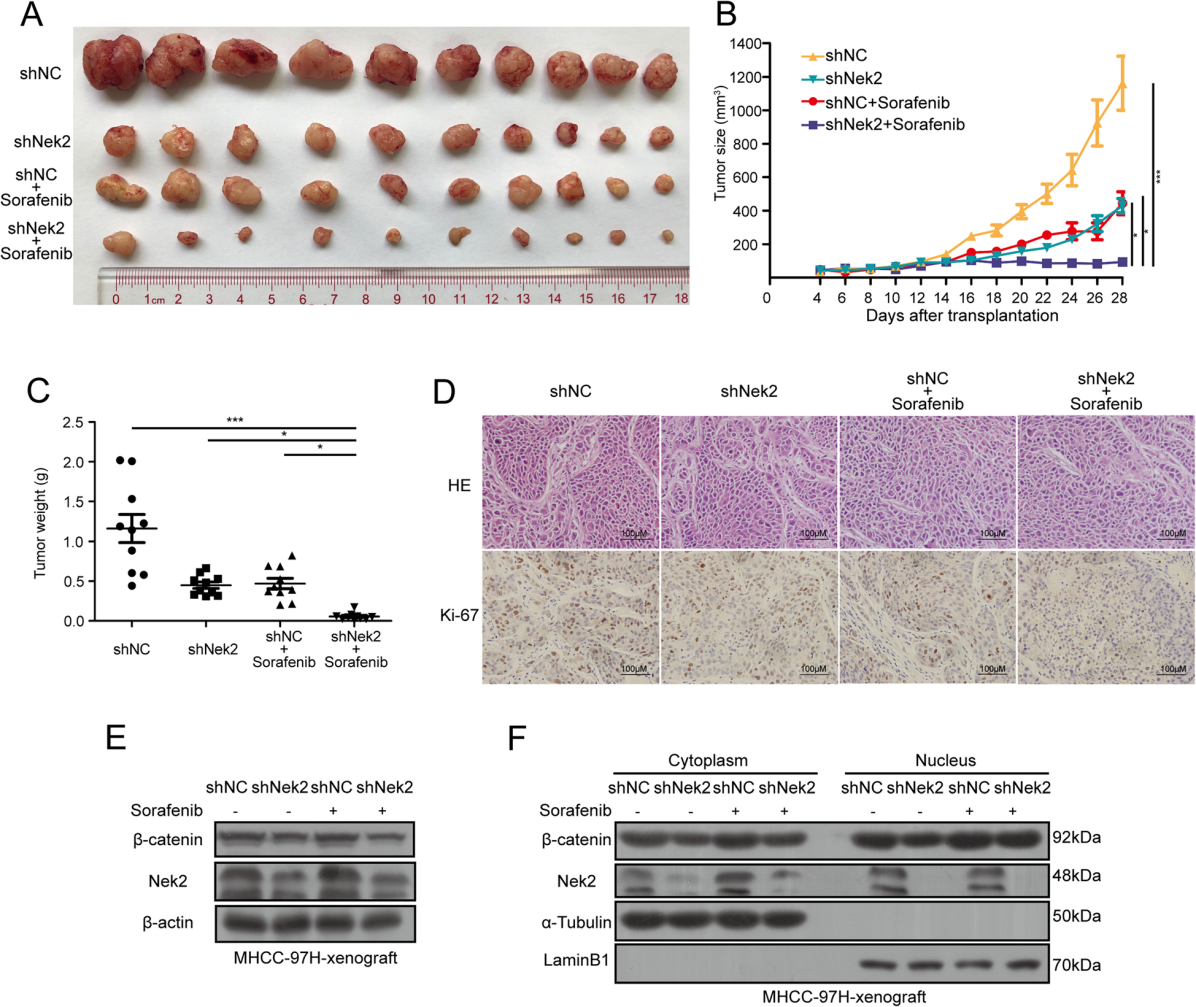
Nek2 knockdown promoted sorafenib efficacy in vivo. **a**. Morphologies of collected tumors in each group. **b**. Curves of tumor growth in each group. **c**. Tumor weights were measured after collection. **d**. HE-stain (upper panel) and immunohistochemical stain for Ki-67 (bottom panel) using xenograft tumor samples from each group. d. Xenograft tumors were extracted for total protein and analyzed with western blotting assay. e. Xenograft tumors were extracted for cytoplasm and nucleus protein and analyzed with western blotting assay. Data were presented as mean ± SEM, *P<0.05; **P<0.01; ***P<0.001

We next attempted to further confirm the role of Nek2 in sorafenib resistance in vivo. We used stable knockdown-MHCC-97H cells to establish xenograft tumor models. We observed that either single knockdown of Nek2 or sorafenib treatment could inhibit tumor growth; however, the combination of Nek2 knockdown and sorafenib treatment resulted in the most significant inhibition of tumor growth (Fig. 5a, b). An analysis of tumor weights at the end of the experiment also indicated that Nek2 knockdown could significantly improve the efficacy of sorafenib treatment (Fig. 5c). We also used immunohistochemical analysis to detect the expression of Ki-67 in the xenograft tumors. Results showed that the combination group had the lowest expression of Ki-67 (Fig. 5d). By analyzing protein extracted from xenograft tumors, we also showed that Nek2 knockdown could decrease the levels of β-catenin (Fig. 5e). Further analysis revealed that Nek2 suppression also decreased the distribution of β-catenin in the nucleus (Fig. 5f), indicating that Nek2 could overcome sorafenib resistance via the regulation of β-catenin in vivo. Together, these results show that Nek2 knockdown mitigates sorafenib resistance and improves the anti-tumor effects of this drug.

We also intended to confirm this result with Nek2 inhibitor. All Commercial inhibitors of Nek2 have focused on the interaction between Nek2 and Hec1, a member of the kinetochore that regulates the spindle checkpoint, as disrupting this interaction leads to the degradation of Nek2. We chose TAI-1, the only one that has been used in HCC cell lines [20]. First we tested the effect of TAI-1 in MHCC-97H. The results showed that TAI-1 decreased the protein level of Nek2 in HCC cell line (Additional file 6: Figure S6a). Next we test the effect of TAI-1 in vivo. The results showed that sorafenib and combination treatment both inhibited the growth of tumor, while TAI-1 alone failed to make a significant effect compared to the control group (Additional file 6: Figure S6b, c). We noticed that the difference between combination group and sorafenib alone group was not significant. Weight of the xenograft tumors also showed that the combination of sorafenib and TAI-1 failed to make a significant decrease compared to the sorafenib group (Additional file 6: Figure S6d).

### Nek2 is associated with HCC progression and poor prognosis

**Fig. 6:**
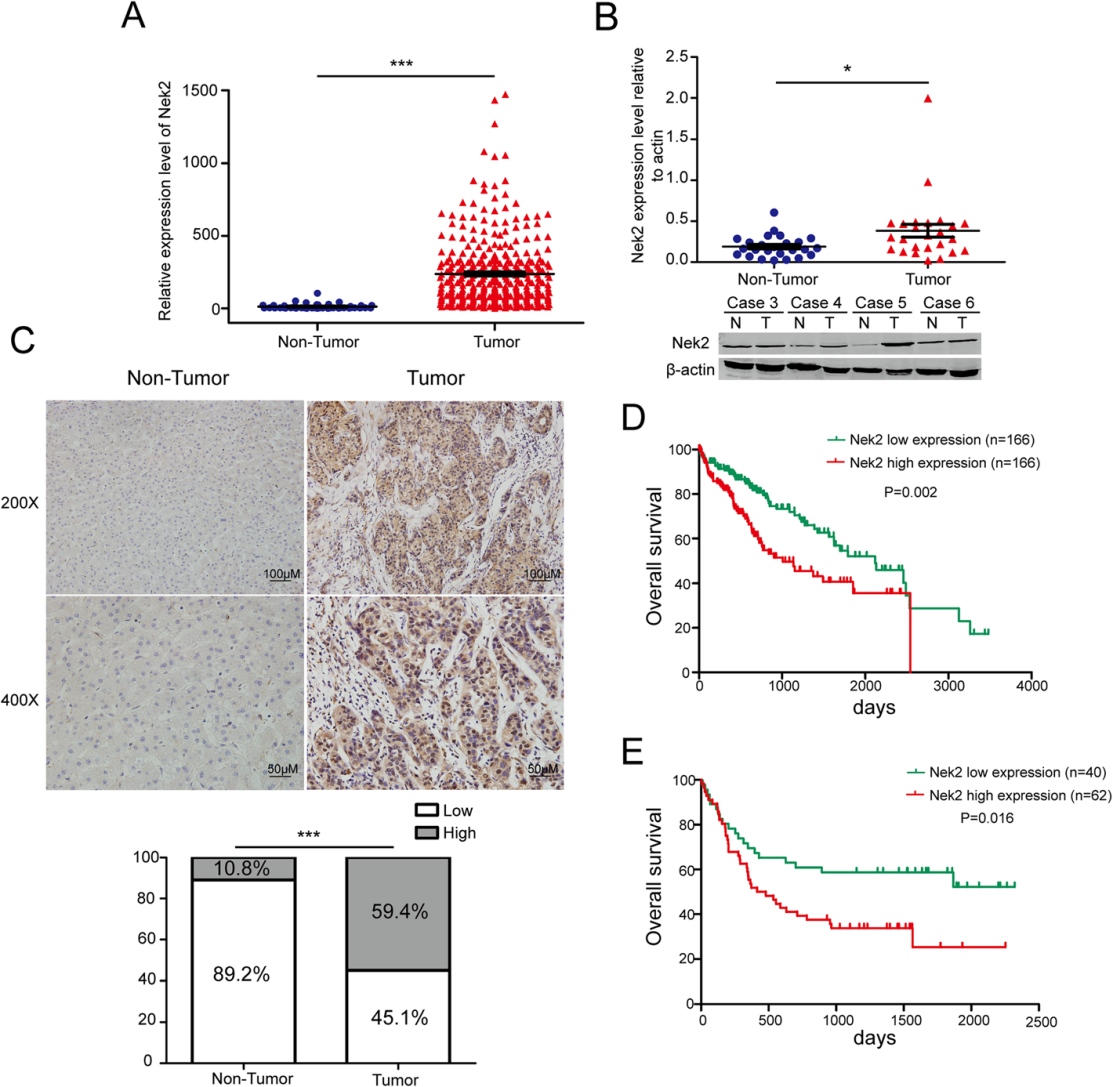
Nek2 expression level was elevated in HCC patients and associated with prognosis. **a**. A dot plot showed the mRNA expression levels of Nek2 in HCC tissues from TCGA cohort. **b**. (Upper panel) A dot plot showed the protein levels of 29 pairs of HCC tissues. (Bottom panel) Four pairs of HCC tissues displayed as representative examples. **c**. (Upper panel) A representative results of immunohistochemistry showing the expression of Nek2 in tumor tissues and paired non-tumor tissue. (Bottom panel) Statistical analysis for 102 paired non-tumor and tumor paraffin-embedded HCC tissues. **d**, **e**. Kaplan-Meier survival analysis for overall survival of TCGA cohort (**d**) and 102 pairs of paraffin-embedded HCC tissues (**e**) according to the expression level of Nek2. Data were presented as mean ± SEM, *P<0.05; **P<0.01; ***P<0.001

To determine the role of Nek2 in HCC, we first examined its mRNA levels using the TCGA cohort and the results showed that levels were significantly higher in tumors (Fig. 6a). We also analyzed 29 pairs of HCC tissues, comparing them to corresponding non-tumor tissues, by western blotting and showed that there was a significant increase in Nek2 protein levels (Fig. 6b, Additional file 7: Figure S7a). To further validate the dysregulation of Nek2, we detected its expression in 102 paraffin-embedded HCC tissues and paired non-tumor tissues by immunohistochemistry. The percentage of HCC tissues expressing high levels of Nek2 was higher than that with non-tumor tissues (Fig. 6c). Clinicopathological characteristics and the correlations between these paremeters and Nek2 expression are shown in Additional file 8: Table S1 and Additional file 9: Table S2. Notably, high expression of Nek2 in HCC was significantly correlated with alpha-fetoprotein (AFP, *P* = 0.028), tumor size (*P* = 0.02), Barcelona Clinic Liver Cancer stage (BCLC stage, *P* = 0.031), and local relapse (*P* = 0.034). As is known, sorafenib is the first-line treatment for BCLC advanced-stage HCC patients and our data showed that high Nek2 levels are correlated with advanced stage. We further analyzed the correlation between Nek2 expression and tumor pathologic stage based on the TCGA cohort and found a significant difference in Nek2 levels between the low/intermediate-grade and high -grade groups (Additional file 7: Figure S7b).

Survival analysis also showed that based on the TCGA cohort and 102 HCC tissues, high Nek2 expression was associated with shorter overall survival and recurrence-free survival, compared to those in patients with low Nek2 expression (Fig. 6d, e and Additional file 7: Figure S7c, d). Furthermore, univariate and multivariate analyses of overall survival based on 102 HCC patients and Cox regression analysis showed that Nek2 (95% CI: 1.048–5.442; *P* = 0.038) was an independent prognostic indicator for HCC (Additional file 10: Table S3).

Taken together, this study provides evidence that Nek2 induces sorafenib resistance by interacting with β-catenin and activating the Wnt pathway in HCC.

## Discussion

This study was the first to identify the role of Nek2 in HCC with regard to sorafenib resistance. We observed that the overexpression of Nek2 inhibited apoptosis and cell growth inhibition induced by sorafenib treatment, whereas silencing this gene significantly enhanced sorafenib-induced apoptosis and cell growth inhibition. Consistently, a xenograft tumor model showed that the combination of Nek2 knockdown and sorafenib treatment could significantly inhibit tumor growth compared to that in the control, single knockdown, or sorafenib treatment only groups. Data from both the TCGA cohort and HCC patient samples confirmed that Nek2 is up-regulated in HCC samples, as compared to levels in non-tumor samples, and that higher expression is related to lower overall and recurrence-free survival. Interestingly, correlation analysis indicated a relationship between high levels of Nek2 and advanced HCC stage. Given that sorafenib is the first-line treatment for advanced-stage HCC patients [21], our findings suggest that inhibiting Nek2 might be an ideal strategy to improve the efficacy of sorafenib in HCC.

Previous studies have demonstrated the regulation of β-catenin by Nek2 at mitotic centrosomes, but few studies have also shown a possible correlation between Nek2 and β-catenin in cancer [22, 23]. Further, there is no research on the detailed regulatory mechanism associated with Nek2 and β-catenin in cancer. In our study, we elaborated upon the mechanism through which Nek2 induces sorafenib resistance and found that this occurs via the regulation of β-catenin. Our results also showed that Nek2 interacts with β-catenin and regulates its ubiquitination. The kinase activity of Nek2 was also found to be crucial for the interaction between Nek2 and β-catenin.

Some other studies have shown that Nek2 is upregulated in multiple types of cancer. It was also identified as a gene associated with chromosomal instability that induces chemo-resistance through the activation of efflux pumps in myeloma [24]. In ovarian cancer, Nek2 induces drug resistance by regulating the cell cycle and microtubules [25]. These studies and our results show that Nek2 might contribute to anti-tumor treatment resistance in different types of tumors via different mechanisms.

One study on glioblastoma showed that Nek2 protects EZH2 from ubiquitination-dependent protein degradation and its expression was found to be increased in recurrent tumors [26]. In that study, a specific Nek2 kinase inhibitor that could inhibit tumor growth alone or when combined with radiotherapy was designed. All commercially-available inhibitors of Nek2 have focused on the interaction between Nek2 and Hec1, a member of the kinetochore that regulates the spindle checkpoint, as disrupting this interaction leads to the degradation of Nek2 [27–29]. In the study mention above, a specific Nek2 kinase inhibitor more efficiently inhibited tumor growth than Hec1/Nek2 inhibitors did [26], suggesting that selective kinase inhibitors of Nek2 might be more beneficial for cancer treatment than Hec1-related inhibitors.

In our study, the results of TAI-1, the Hec1/Nek2 inhibitor was not ideal as Nek2 knockdown. The possible explanation was that TAI-1 was not a specific Nek2 inhibitor, the primary target was Hec1 and the side effect was down-regulation of Nek2. It could triggered other unknown pathway that weaken the effect of Nek2 down-regulation. Our results and studies mentioned above all calls for development of specific Nek2 inhibitor.

Our results and those of previous studies show that Nek2 localizes to both the cytoplasm and nucleus. In our study, the mechanism through which Nek2 induces sorafenib resistance was found to be through the regulation of β-catenin ubiquitination in the cytoplasm. Using a nuclear localization-deficient Nek2 mutant, we confirmed that when Nek2 lost this ability, it could still up-regulate β-catenin, but that nuclear translocation and downstream target gene transcription were suppressed. This suggested that Nek2 localization to the nucleus is also involved in the regulation of β-catenin; however, this mechanism requires further study.

## Conclusions

Collectively, our study showed that Nek2 binds β-catenin and regulates its ubiquitination and localization. Nek2/β-catenin can also contribute to sorafenib resistance in HCC, and thus represents a potential therapeutic target to improve HCC patient response to sorafenib, especially in those resistant to this agent. Our work also calls for the development of a selective kinase inhibitor to better explore the function of Nek2 in cancer.

## Additional files

Additional file 1: **Figure S1.** β-catenin suppressed cells apoptosis and growth inhibition induced by sorafenib in HCC cell lines. a. SMMC-7721, MHCC-97H and SK-Hep1 HCC cell lines were treated with sorafenib for 24 h and analyzed by western blotting assay. b. SK-Hep1 cells were transfected with siRNA for scramble or β-catenin for 24 h and sorafenib (5 μM) treatment for another 24 h before western blotting assay. c. CCK-8 assays were performed to detect the growth inhibition induced by sorafenib on SK-Hep1 transfected with si-β-catenin. d. (Left panel) SK-Hep1 cells transfected with si-β-catenin were treated with sorafenib and cells were analyzed by flow cytometry. (Right panel) Columns, representing the total percentage of Q2 and Q4, were the average of three independent experiments. e. Dose-dependent effects of sorafenib on the viability of SK-Hep1 with scramble or si-β-catenin. Data were presented as mean ± SEM, ns, no significance; *P<0.05; **P<0.01; ***P<0.001. (TIF 6335 kb)

Additional file 2: **Figure S2.** Nek2 bond β-catenin and regulated its protein level and nuclear translocation in HCC cell lines. a. MHCC-97H cells were transfected with indicated plasmids with or without sorafenib treatment. Flag-tagged protein (left panel) or β-catenin (right panel) were precipitated and associated protein were monitored with western blotting assay. b. HEK-293 T cells were transfected with indicated plasmids. Flag-tagged protein were precipitated and associated protein were monitored with western blotting assay. c. SK-Hep1 cells, transfected with lentivirus containing shRNA for Nek2 were treated with sorafenib for 24 h and analyzed with western blotting assay. d, e, f. qRT-PCR analysis were performed to detect β-catenin and Wnt pathway downstream target genes mRNA levels of HCC cell lines with Nek2 overexpression or knockdown. g, h. SMMC-7721 and MHCC-97H cells were separately extracted for cytoplasmic and nuclear protein and analyzed by western blotting assay. (TIF 4880 kb)

Additional file 3: **Figure S3.** Nek2 stabilized β-catenin. a. An illustration demonstrated the mutantion plasmids of Nek2. b. SMMC-7721 cells transfected with wild type or NLSm of Nek2 were separately extracted for total, cytoplasmic and nuclear protein and analyzed by western blotting assay. c. MHCC-97H with knockdown of Nek2 were treated with or without MG132 for 6 h and harvested for western blotting assay. d. SK-Hep1 transfected with scramble or si-Nek2 were treated with CHX (10 μM) and cells were collected at indicated timings. e. SMMC-7721 transfected with empty vector or NLSm were treated with CHX (10 μM) and cells were collected at indicated timings. (TIF 2223 kb)

Additional file 4: **Figure S4.** Nek2 induced sorafenib resistance in HCC cell lines. a. SK-Hep1 cells with Nek2 knockdown were treated with sorafenib for 24 h and analyzed with western blotting assay for pro-apoptotic and anti-apoptotic proteins. b. CCK-8 assays were performed to detect the growth inhibition of sorafenib on SK-Hep1 with Nek2 knockdown. c. (Left panel) SK-Hep1 cells with Nek2 knockdown were treated with sorafenib and cells were analyzed by flow cytometry. (Right panel) Columns, representing the total percentage of Q2 and Q4, were the average of three independent experiments. Data were presented as mean ± SEM, ns, no significance; *P<0.05; **P<0.01; ***P<0.001. (TIF 2539 kb)

Additional file 5: **Figure S5.** Nek2 induced sorafenib resistance through β-catenin in HCC cell lines. a. SK-Hep1 transfected with different combinations of shRNA Nek2 and adenovirus-β-catenin were treated with or without sorafenib and the level of pro-apoptotic and anti-apoptotic proteins were analyzed using western blotting assay. b. (Left panel) Flow cytometry was used to detect the apoptosis of different combinations indicated in SK-Hep1. (Right panel) Columns, representing the total percentage of Q2 and Q4, were the average of three independent experiments. Data were presented as mean ± SD, ns, no significance; *P<0.05; **P<0.01; ***P<0.001. c. Dose-dependent effects of sorafenib on the viability of SK-Hep1 with different levels of Nek2 and β-catenin. (TIF 3515 kb)

Additional file 6: **Figure S6.** Nek2 inhibitor TAI-1 failed to improve the efficiency of sorafenib in vivo. Twenty nude mice were randomly divided into 4 groups (*n* = 5 per group). When the average volume of tumor reached 100mm^3^, Mice in each group received indicated treatments. In sorafenib treatment groups, mice were given sorafenib 30 mg/kg/d by gavage for 14 days before sacrifice. TAI-1 was intraperitoneal injection at a dose of 20 mg/kg/day for 14 days, synchronized with sorafenib. Tumors were measured every two days since the fourth day after injection. a. MHCC-97H was treated with TAI-1 and the protein levels were measured with western blotting. b. Morphologies of collected tumors in each group. c. Curves of tumor growth in each group. d. Tumor weights were measured after collection of xenograft tumors. (TIF 2062 kb)

Additional file 7: **Figure S7.** Nek2 was up-regulated in high grade of tumor and correlated with poor recurrence-free survival. a.Expression level of Nek2 in 29 paired HCC samples. b. Nek2 expression level of low/intermediate and high differentiated tumor grade using TCGA cohort. c, d. Kaplan-Meier survival analysis for recurrence-free survival of TCGA cohort (c) and 102 pairs of paraffin-embedded HCC tissues (d) according to the expression level of Nek2. Data were presented as mean ± SEM, *P<0.05; **P<0.01; ***P<0.001. (TIF 2759 kb)

Additional file 8: **Table S1.** Clinicopathological characteristics of 102 HCC patients. (DOC 38 kb)

Additional file 9: **Table S2.** Correlation between Nek2 expression and HCC clinicopathologic features. (DOCX 16 kb)

Additional file 10: **Table S3.** Univariate and multivariate analyses of OS in 102 HCC patients by Cox regression analysis. (DOCX 14 kb)

## Data Availability

Gene expression data (GSE62813 and GSE74666 profiling data) were downloaded as raw signals from Gene Expression Omnibus (http://www.ncbi.nlm.nih.gov/geo). The cancer genome atlas program data was downloaded from National Cancer Institute (https://www.cancer.gov/about-nci/organization/ccg/research/structural-genomics/tcga).

## Abbreviations

AFP: alpha-fetoprotein
BCLC: Barcelona Clinic Liver Cancer
HCC: hepatocellular carcinoma
Nek2: NIMA-related kinase 2
NLSm: nuclear localization sequence mutant of Nek2
