# Supplementary figures and images for: Nek2 augments sorafenib resistance by regulating the ubiquitination and localization of β-catenin in hepatocellular carcinoma
Source: J Exp Clin Cancer Res. 2019 Jul 18;38:316. doi: 10.1186/s13046-019-1311-z (PMC6639974; doi:10.1186/s13046-019-1311-z)

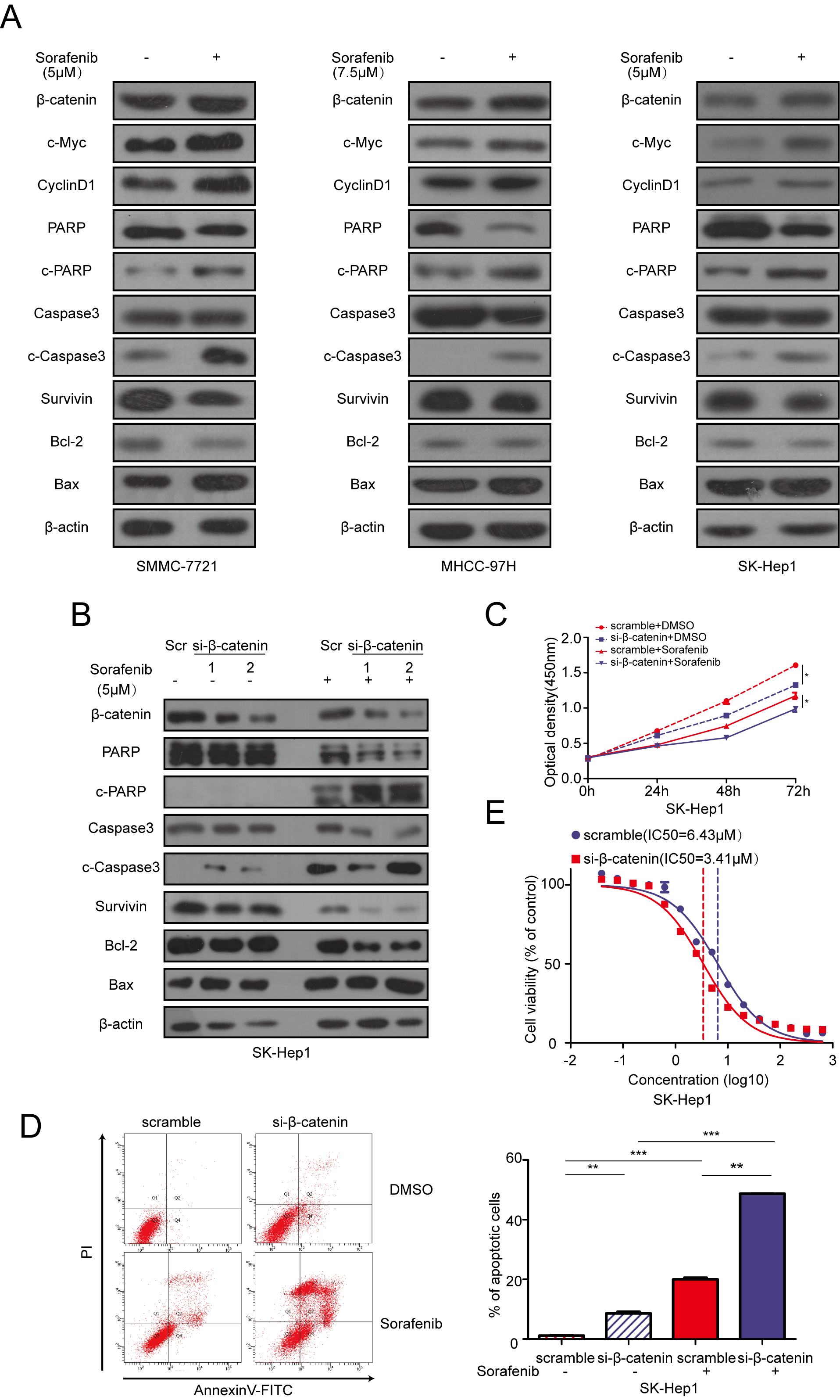

Supplement: Supplementary file 1 — Figure S1. β-catenin suppressed cells apoptosis and growth inhibition induced by sorafenib in HCC cell lines. a. SMMC-7721, MHCC-97H and SK-Hep1 HCC cell lines were treated with sorafenib for 24 h and analyzed by western blotting assay. b. SK-Hep1 cells were transfected with siRNA for scramble or β-catenin for 24 h and sorafenib (5 μM) treatment for another 24 h before western blotting assay. c. CCK-8 assays were performed to detect the growth inhibition induced by sorafenib on SK-Hep1 transfected with si-β-catenin. d. (Left panel) SK-Hep1 cells transfected with si-β-catenin were treated with sorafenib and cells were analyzed by flow cytometry. (Right panel) Columns, representing the total percentage of Q2 and Q4, were the average of three independent experiments. e. Dose-dependent effects of sorafenib on the viability of SK-Hep1 with scramble or si-β-catenin. Data were presented as mean ± SEM, ns, no significance; *P<0.05; **P<0.01; ***P<0.001. (TIF 6335 kb) [file 13046_2019_1311_MOESM1_ESM.tif]

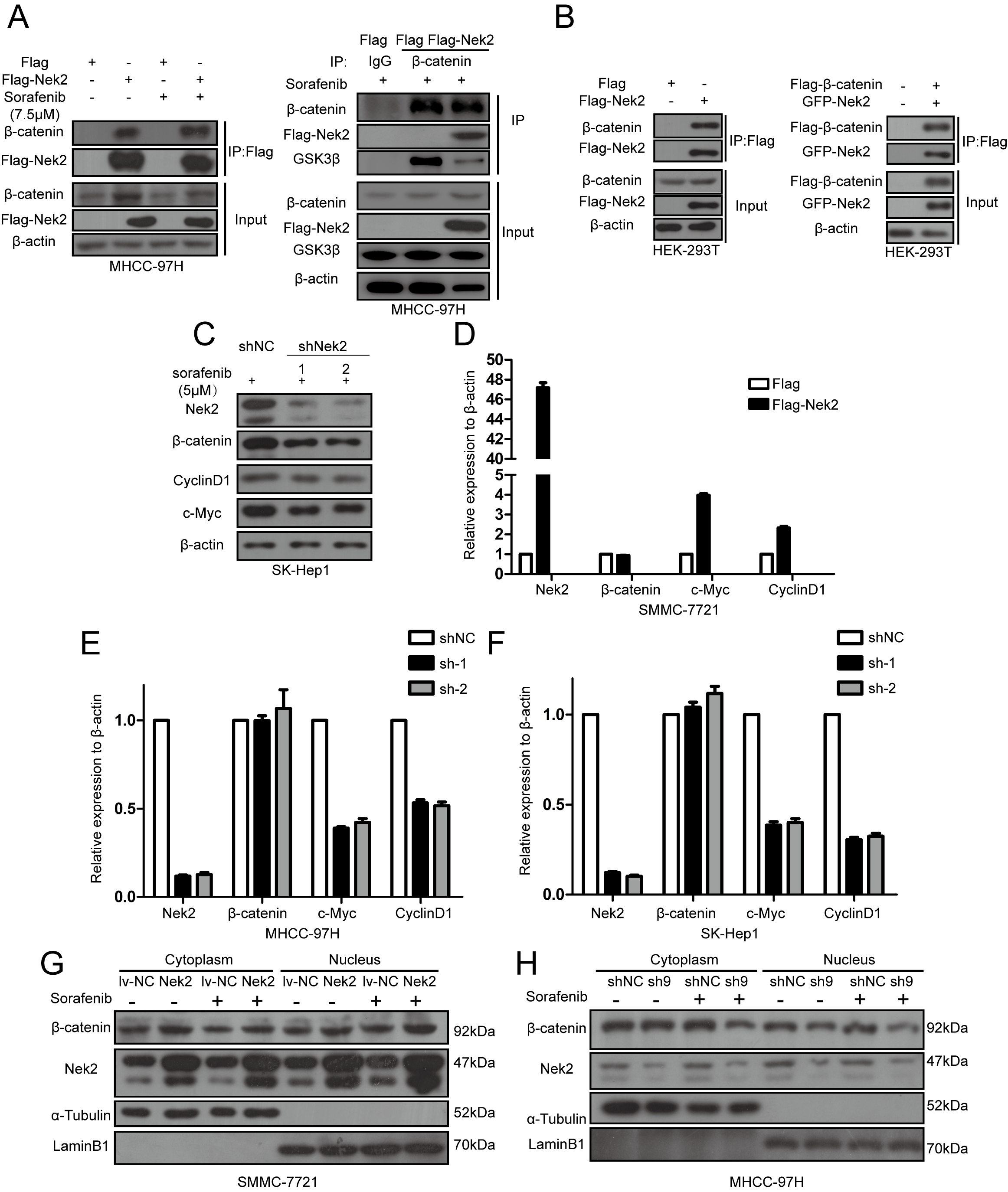

Supplement: Supplementary file 2 — Figure S2. Nek2 bond β-catenin and regulated its protein level and nuclear translocation in HCC cell lines. a. MHCC-97H cells were transfected with indicated plasmids with or without sorafenib treatment. Flag-tagged protein (left panel) or β-catenin (right panel) were precipitated and associated protein were monitored with western blotting assay. b. HEK-293 T cells were transfected with indicated plasmids. Flag-tagged protein were precipitated and associated protein were monitored with western blotting assay. c. SK-Hep1 cells, transfected with lentivirus containing shRNA for Nek2 were treated with sorafenib for 24 h and analyzed with western blotting assay. d, e, f. qRT-PCR analysis were performed to detect β-catenin and Wnt pathway downstream target genes mRNA levels of HCC cell lines with Nek2 overexpression or knockdown. g, h. SMMC-7721 and MHCC-97H cells were separately extracted for cytoplasmic and nuclear protein and analyzed by western blotting assay. (TIF 4880 kb) [file 13046_2019_1311_MOESM2_ESM.tif]

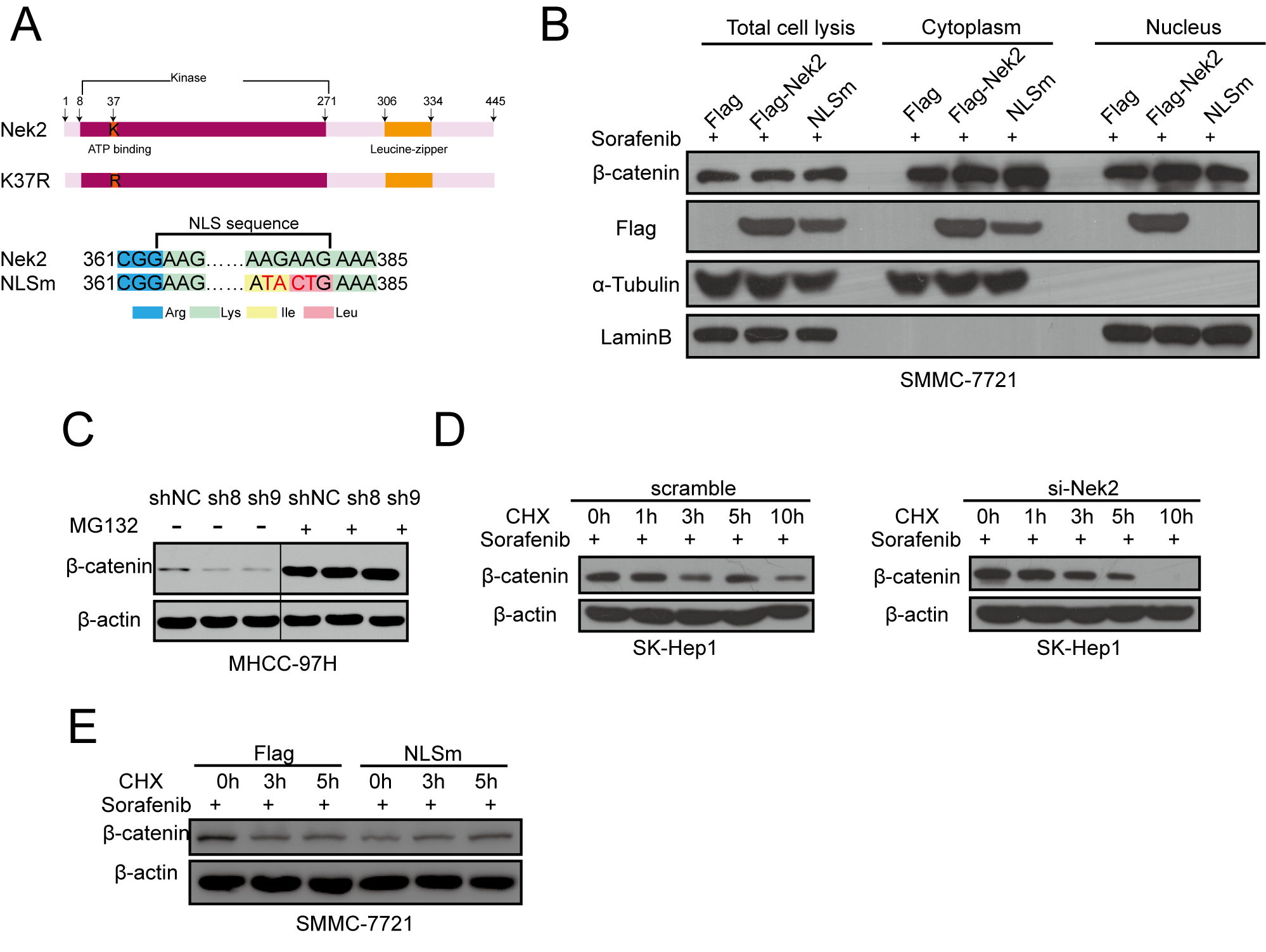

Supplement: Supplementary file 3 — Figure S3. Nek2 stabilized β-catenin. a. An illustration demonstrated the mutantion plasmids of Nek2. b. SMMC-7721 cells transfected with wild type or NLSm of Nek2 were separately extracted for total, cytoplasmic and nuclear protein and analyzed by western blotting assay. c. MHCC-97H with knockdown of Nek2 were treated with or without MG132 for 6 h and harvested for western blotting assay. d. SK-Hep1 transfected with scramble or si-Nek2 were treated with CHX (10 μM) and cells were collected at indicated timings. e. SMMC-7721 transfected with empty vector or NLSm were treated with CHX (10 μM) and cells were collected at indicated timings. (TIF 2223 kb) [file 13046_2019_1311_MOESM3_ESM.tif]

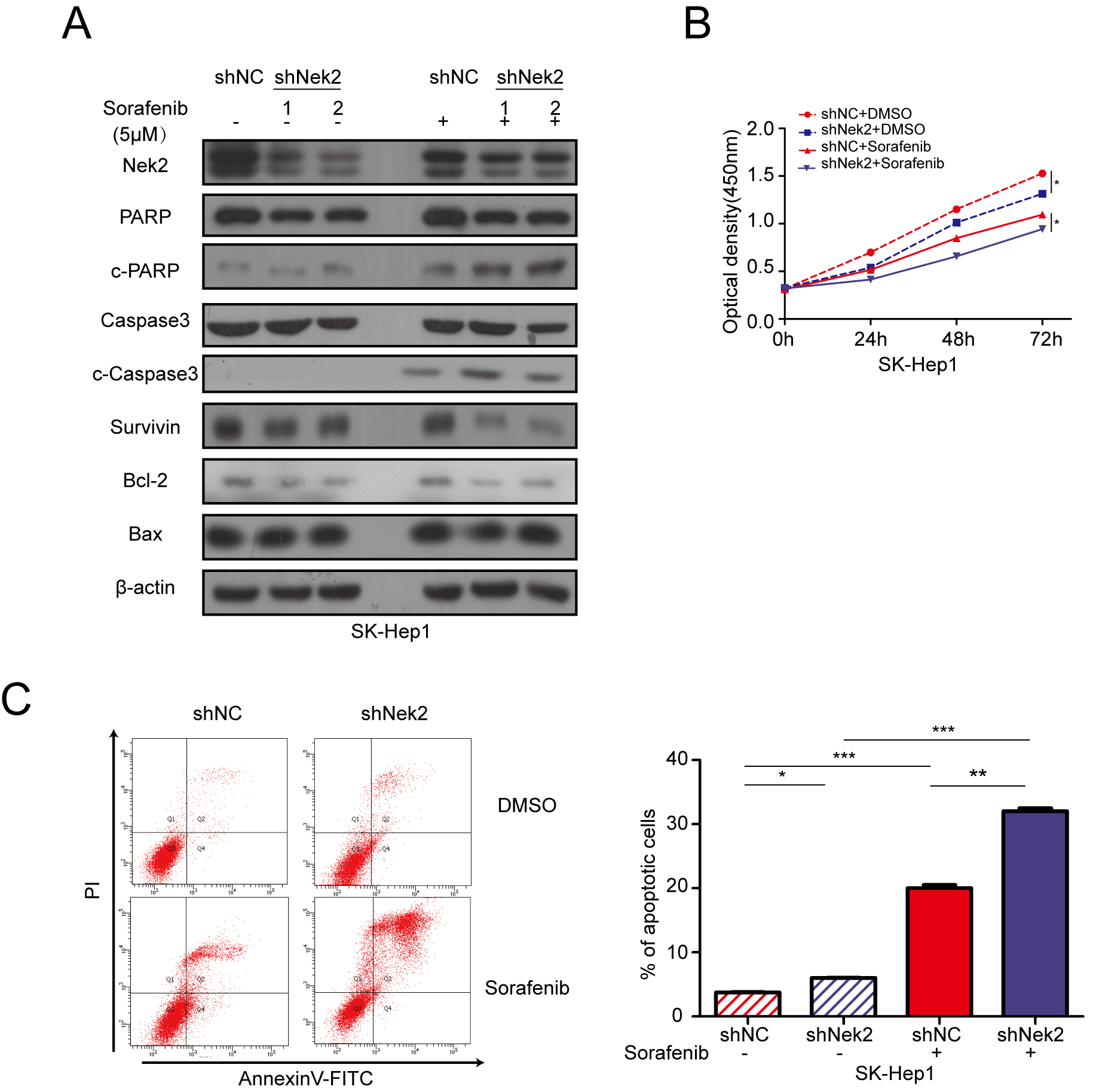

Supplement: Supplementary file 4 — Figure S4. Nek2 induced sorafenib resistance in HCC cell lines. a. SK-Hep1 cells with Nek2 knockdown were treated with sorafenib for 24 h and analyzed with western blotting assay for pro-apoptotic and anti-apoptotic proteins. b. CCK-8 assays were performed to detect the growth inhibition of sorafenib on SK-Hep1 with Nek2 knockdown. c. (Left panel) SK-Hep1 cells with Nek2 knockdown were treated with sorafenib and cells were analyzed by flow cytometry. (Right panel) Columns, representing the total percentage of Q2 and Q4, were the average of three independent experiments. Data were presented as mean ± SEM, ns, no significance; *P<0.05; **P<0.01; ***P<0.001. (TIF 2539 kb) [file 13046_2019_1311_MOESM4_ESM.tif]

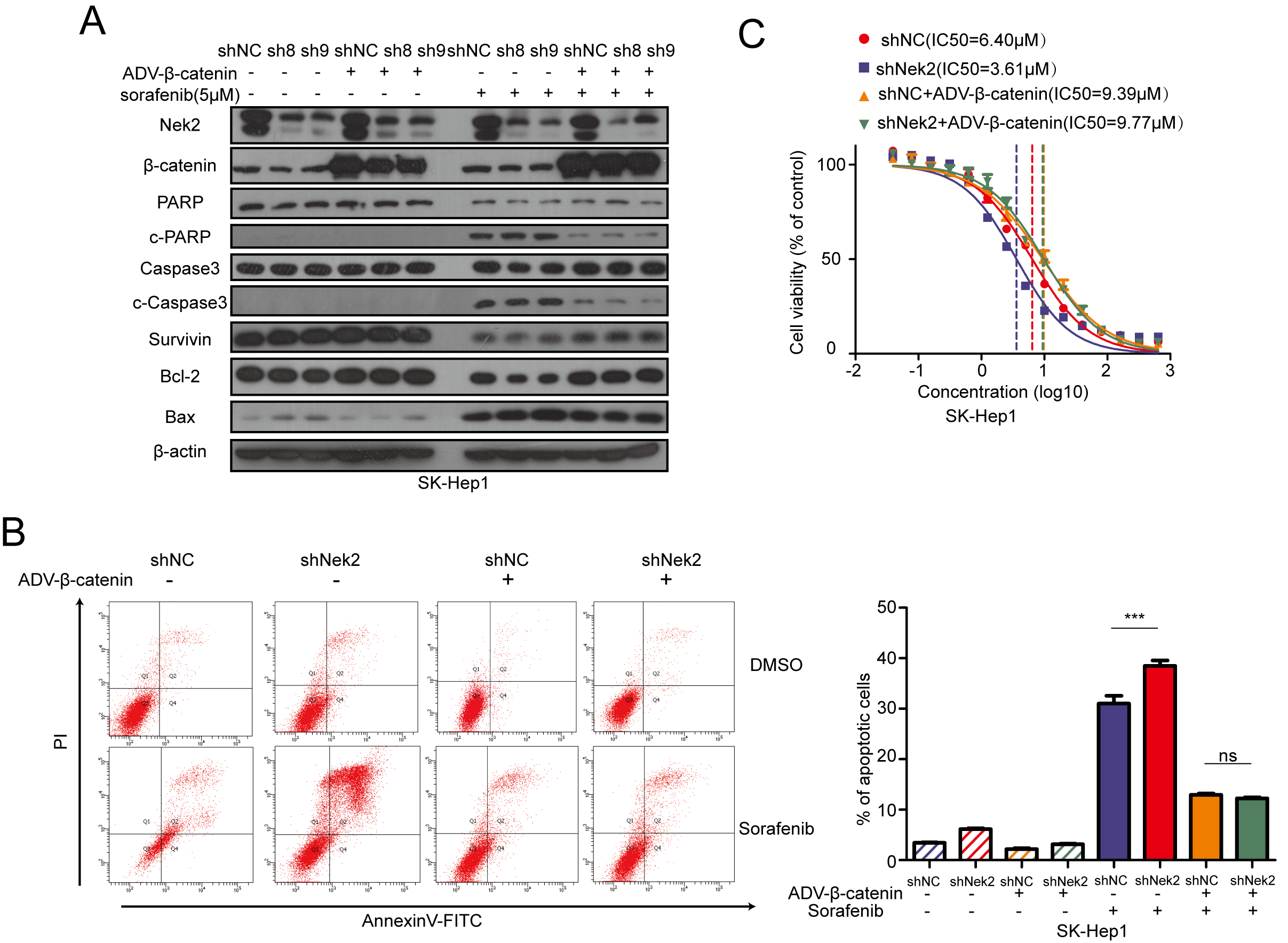

Supplement: Supplementary file 5 — Figure S5. Nek2 induced sorafenib resistance through β-catenin in HCC cell lines. a. SK-Hep1 transfected with different combinations of shRNA Nek2 and adenovirus-β-catenin were treated with or without sorafenib and the level of pro-apoptotic and anti-apoptotic proteins were analyzed using western blotting assay. b. (Left panel) Flow cytometry was used to detect the apoptosis of different combinations indicated in SK-Hep1. (Right panel) Columns, representing the total percentage of Q2 and Q4, were the average of three independent experiments. Data were presented as mean ± SD, ns, no significance; *P<0.05; **P<0.01; ***P<0.001. c. Dose-dependent effects of sorafenib on the viability of SK-Hep1 with different levels of Nek2 and β-catenin. (TIF 3515 kb) [file 13046_2019_1311_MOESM5_ESM.tif]

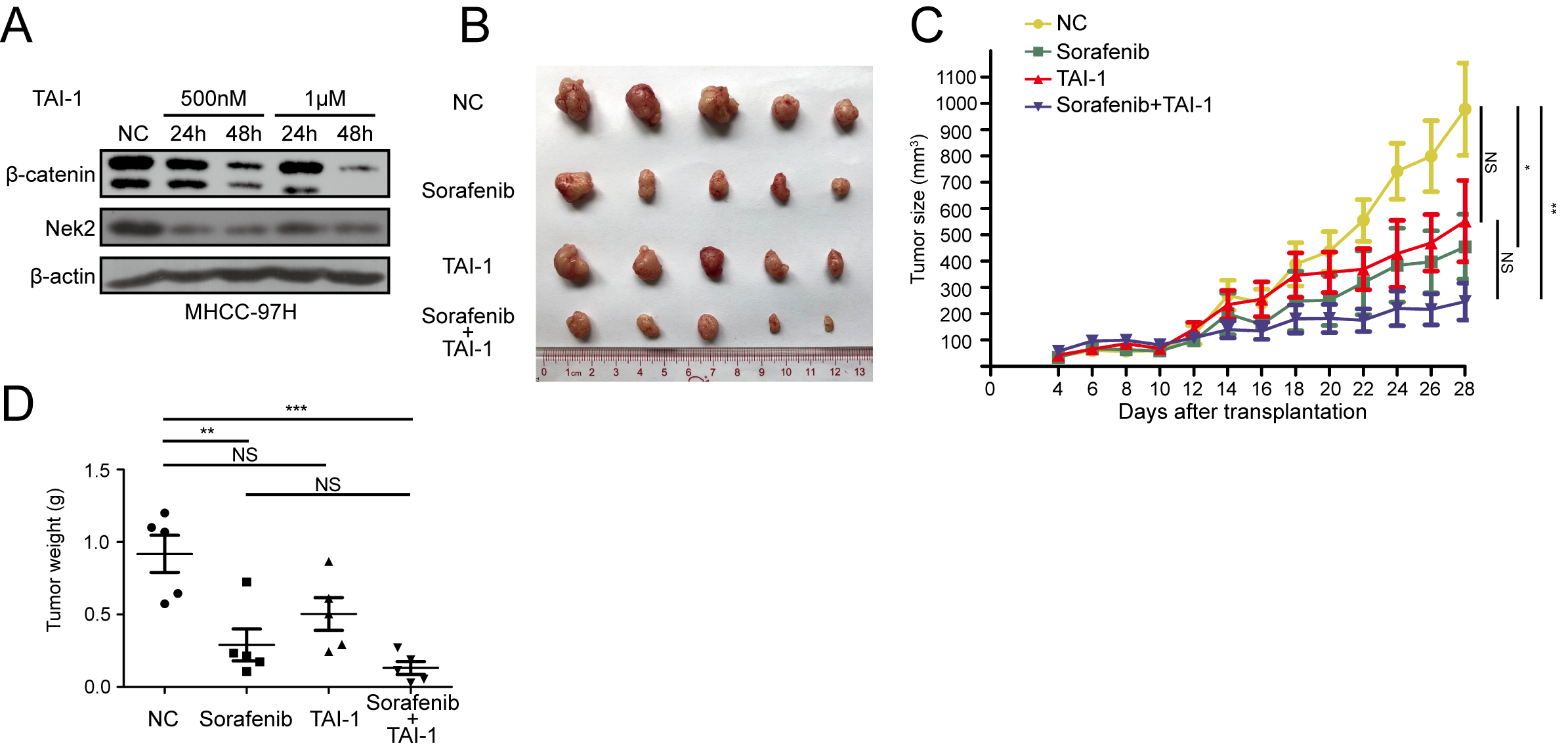

Supplement: Supplementary file 6 — Figure S6. Nek2 inhibitor TAI-1 failed to improve the efficiency of sorafenib in vivo. Twenty nude mice were randomly divided into 4 groups (n = 5 per group). When the average volume of tumor reached 100mm3, Mice in each group received indicated treatments. In sorafenib treatment groups, mice were given sorafenib 30 mg/kg/d by gavage for 14 days before sacrifice. TAI-1 was intraperitoneal injection at a dose of 20 mg/kg/day for 14 days, synchronized with sorafenib. Tumors were measured every two days since the fourth day after injection. a. MHCC-97H was treated with TAI-1 and the protein levels were measured with western blotting. b. Morphologies of collected tumors in each group. c. Curves of tumor growth in each group. d. Tumor weights were measured after collection of xenograft tumors. (TIF 2062 kb) [file 13046_2019_1311_MOESM6_ESM.tif]

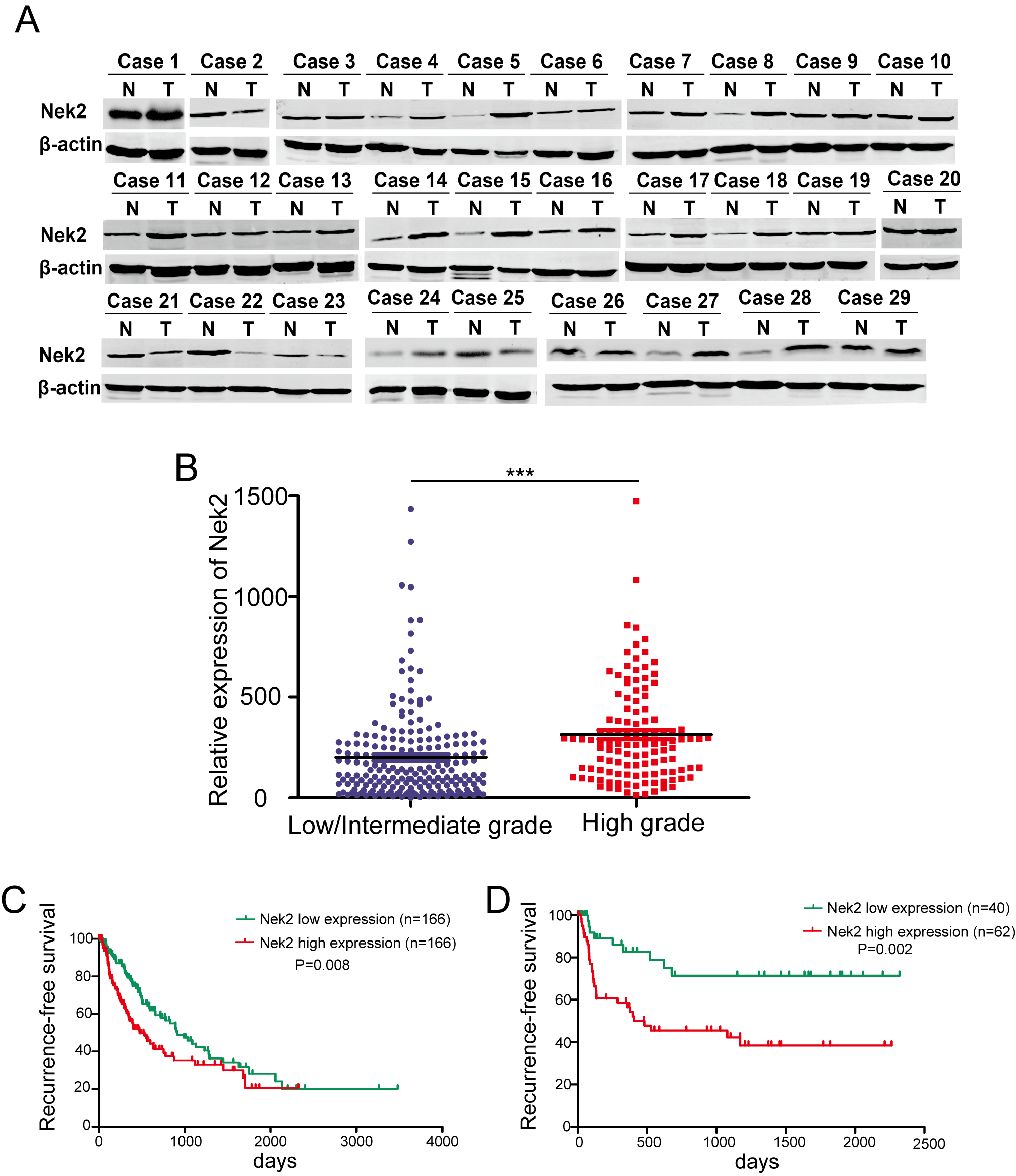

Supplement: Supplementary file 7 — Figure S7. Nek2 was up-regulated in high grade of tumor and correlated with poor recurrence-free survival. a.Expression level of Nek2 in 29 paired HCC samples. b. Nek2 expression level of low/intermediate and high differentiated tumor grade using TCGA cohort. c, d. Kaplan-Meier survival analysis for recurrence-free survival of TCGA cohort (c) and 102 pairs of paraffin-embedded HCC tissues (d) according to the expression level of Nek2. Data were presented as mean ± SEM, *P<0.05; **P<0.01; ***P<0.001. (TIF 2759 kb) [file 13046_2019_1311_MOESM7_ESM.tif]
