# Supplementary material for: Nek2 augments sorafenib resistance by regulating the ubiquitination and localization of β-catenin in hepatocellular carcinoma
Source: J Exp Clin Cancer Res. 2019 Jul 18;38:316. doi: 10.1186/s13046-019-1311-z (PMC6639974; doi:10.1186/s13046-019-1311-z)
Supplement: Supplementary file 8 — Table S1. Clinicopathological characteristics of 102 HCC patients. (DOC 38 kb) [file 13046_2019_1311_MOESM8_ESM.doc]

**Supplementary Table S1. Clinicopathological characteristics of 102 HCC patients**

| Feature | N（%） |
| --- | --- |
| Age(years) |  |
| ≤55  >55 | 59 (57.8)  43 (42.2) |
| Gender |  |
| Male  Female | 82 (80.4)  20 (19.6) |
| Tumor(cm)  ≤3  >3  Pathological grade  I-II  III-IV  BCLC stage  A+B  C+D  Portal vein tumor thrombus  No  Yes | 28 (27.5)  74 (72.5)  36 (35.3)  66 (64.7)  76 (74.5)  26 (25.5)  78 (76.5)  24 (23.5) |
| AFP(ng/ml) |  |
| ≤20  >20 | 34 (33.3)  68 (66.7) |
| Liver cirrhosis |  |
| Yes  No | 70 (68.6)  32 (31.4) |
| Metastasis |  |
| Yes  No | 9 (8.8)  93 (91.2) |
| Relapse |  |
| Yes  No  No. tumor  Solitary  Multiple  Envelop  With  Without | 40 (39.2)  62 (60.8)  75（73.5）  27（26.5）  58 (56.9)  44 (43.1) |

**Abbreviations:** AFP, alpha-fetoprotein; BCLC, Barcelona Clinic Liver Cancer.
