# Supplementary material for: Nek2 augments sorafenib resistance by regulating the ubiquitination and localization of β-catenin in hepatocellular carcinoma
Source: J Exp Clin Cancer Res. 2019 Jul 18;38:316. doi: 10.1186/s13046-019-1311-z (PMC6639974; doi:10.1186/s13046-019-1311-z)
Supplement: Supplementary file 9 — Table S2. Correlation between Nek2 expression and HCC clinicopathologic features. (DOCX 16 kb) [file 13046_2019_1311_MOESM9_ESM.docx]

| **Table 2. Correlation between Nek2 expression and HCC clinicopathologic features** | | | | | | |
| --- | --- | --- | --- | --- | --- | --- |
|  | Nek2 expression | | *P* | |  |  |
|  | Low | High |  |  |  |  |
| Gender  Male  Female | 62  19 | 20  1 | 0.055 |  |  |  |
| Age (years) |  |  | 0.574 |  |  |  |
| ≤55  >55 | 48  33 | 11  10 |  |  |  |  |
| Liver cirrhosis  With  without | 55  26 | 15  6 | 0.486 |  |  |  |
| Encapsulation  With  without | 43  38 | 15  6 | 0.149 |  |  |  |
| AFP (μg/L) |  |  | **0.028*** |  |  |  |
| ≤20  >20  Portal vein tumor thrombus  Yes  No  No. tumor | 20  54  19  62 | 14  14  5  16 | 0.648  0.392 |  |  |  |
| Solitary  multiple  Tumor size (cm)  ≤3  >3  BCLC stage  A+B  C+D  Local relapse  Yes  No  Extrahepatic metastasis  Yes  No | 58  23  18  63  39  7  36  45  5  76 | 17  4  10  11  37  19  4  17  4  17 | **0.02*******  **0.031***  **0.034***  0.065 |  |  |  |

**Abbreviations:** AFP, alpha-fetoprotein; BCLC, Barcelona Clinic Liver Cancer.

*The values in bold had statistically significant differences.
