# Supplementary material for: Nek2 augments sorafenib resistance by regulating the ubiquitination and localization of β-catenin in hepatocellular carcinoma
Source: J Exp Clin Cancer Res. 2019 Jul 18;38:316. doi: 10.1186/s13046-019-1311-z (PMC6639974; doi:10.1186/s13046-019-1311-z)
Supplement: Supplementary file 10 — Table S3. Univariate and multivariate analyses of OS in 102 HCC patients by Cox regression analysis. (DOCX 14 kb) [file 13046_2019_1311_MOESM10_ESM.docx]

**Table 3.** **Univariate and multivariate analyses of OS in 102 HCC patients by Cox regression analysis**

| Variables | Univariate analysis | | |  | Multivariate analysis | | |
| --- | --- | --- | --- | --- | --- | --- | --- |
|  | Hazard ratio | CI (95%) | *P*  value |  | Hazard ratio | CI (95%) | *P*  value |
| gender | 0.606 | 0.331-1.108 | 0.104 |  |  |  |  |
| Age(year) | 1.008 | 0.988-1.029 | 0.423 |  |  |  |  |
| Edmondson Grade | 0.778 | 0.509-1.189 | 0.256 |  |  |  |  |
| metastasis | 1.498 | 0.642-3.495 | 0.349 |  |  |  |  |
| Liver cirrhosis | 0.791 | 0.460-1.395 | 0.396 |  |  |  |  |
| Envelop | 1.055 | 0.625-1.782 | 0.840 |  |  |  |  |
| Tumor number | 1.189 | 0.941-1.502 | 0.146 |  |  |  |  |
| Portal vein tumor thrombus | 2.192 | 1.250-3.845 | **0.006*** |  | 3.396 | 1.879-6.137 | 0.000 |
| BCLC stage | 2.890 | 1.492-5.559 | **0.002*** |  | 2.979 | 1.436-6.180 | 0.003 |
| Relapse | 1.556 | 0.925-2.615 | 0.095 |  |  |  |  |
| Tumor size | 1.460 | 0.812-2.626 | 0.206 |  |  |  |  |
| Nek2 expression | 3.029 | 1.429-6.422 | **0.004*** |  | 2.388 | 1.048-5.442 | 0.038 |

**Abbreviations:** OS, overall survival; BCLC, Barcelona Clinic Liver Cancer; CI, confidence interval; HR, hazard radio.

*The values in bold had statistically significant differences.
